# DAILY INTERACTIONS WITH THE VERY OLD PARENTS AND OLD CHILDREN: CONCORDANCE IN REPORTS AND RELATIONSHIP TYPE

**DOI:** 10.1093/geroni/igz038.3425

**Published:** 2019-11-08

**Authors:** Joohyun Kim, Joohong Min

**Affiliations:** 1 Seoul National University, Seoul, Korea, Republic of; 2 Jeju National University, Jeju, Korea, Republic of

## Abstract

Objectives: Studies have been increased on daily lives of older adults; however, little is known about daily interactions between parents and children, especially both are in their later lives. Utilizing parent-child dyadic data, this study aims to investigate the joint daily interactions of the very old parents and their old children with focus on the relationship type and differences in parent’s and child’s report. Methods: A sample of 105 parents (aged ≥ 80) and 105 children (aged ≥ 65) were interviewed to assess frequency on daily interactions (e.g., contact by phone or email, talking, eating and going out together). Structural Equation Modeling was used to examine differences in amounts of daily interactions by relationship type (e.g., mother-daughter, mother-son and mother-in-law – daughter-in-law). Results: Both parents and children reported high level of daily interactions; however, frequency of watching TV and going out together were relatively lower, compared to other forms of daily interactions. Parents and children showed high concordance in reports; 84% of dyads had the same reports in interactions (absolute mean discrepancy score < 0.1). Mother-daughter relationship reported significantly higher frequency on daily interactions, comparted to mother-son relationship. No significant differences were found between the mother-daughter dyads and the mother-in-law and daughter-in-law dyads. Discussion: Findings suggest that very old parents and old children tend to have high levels of daily interactions, with high concordance in reports. It also highlights its importance of considering relationship type in the dynamic of daily lives and experiences between very old parents and old children.

